# Supplementary material for: Asymmetric Synthesis of Quaternary Hydantoins via a Palladium-Catalyzed Aza-Heck Cyclization
Source: J Am Chem Soc. 2025 Nov 14;147(49):44692–8. doi: 10.1021/jacs.5c16022 (PMC12703750; doi:10.1021/jacs.5c16022)
Supplement: Supplementary file 2 [file ja5c16022_si_002.zip › All NMR FID Files/S16/S16_AllNMR/carbon/pdata/1/email_TDI01074-clean_9_1.pdf]

C13APT256 CDCl3 /opt/nmrdata tdidowu 15

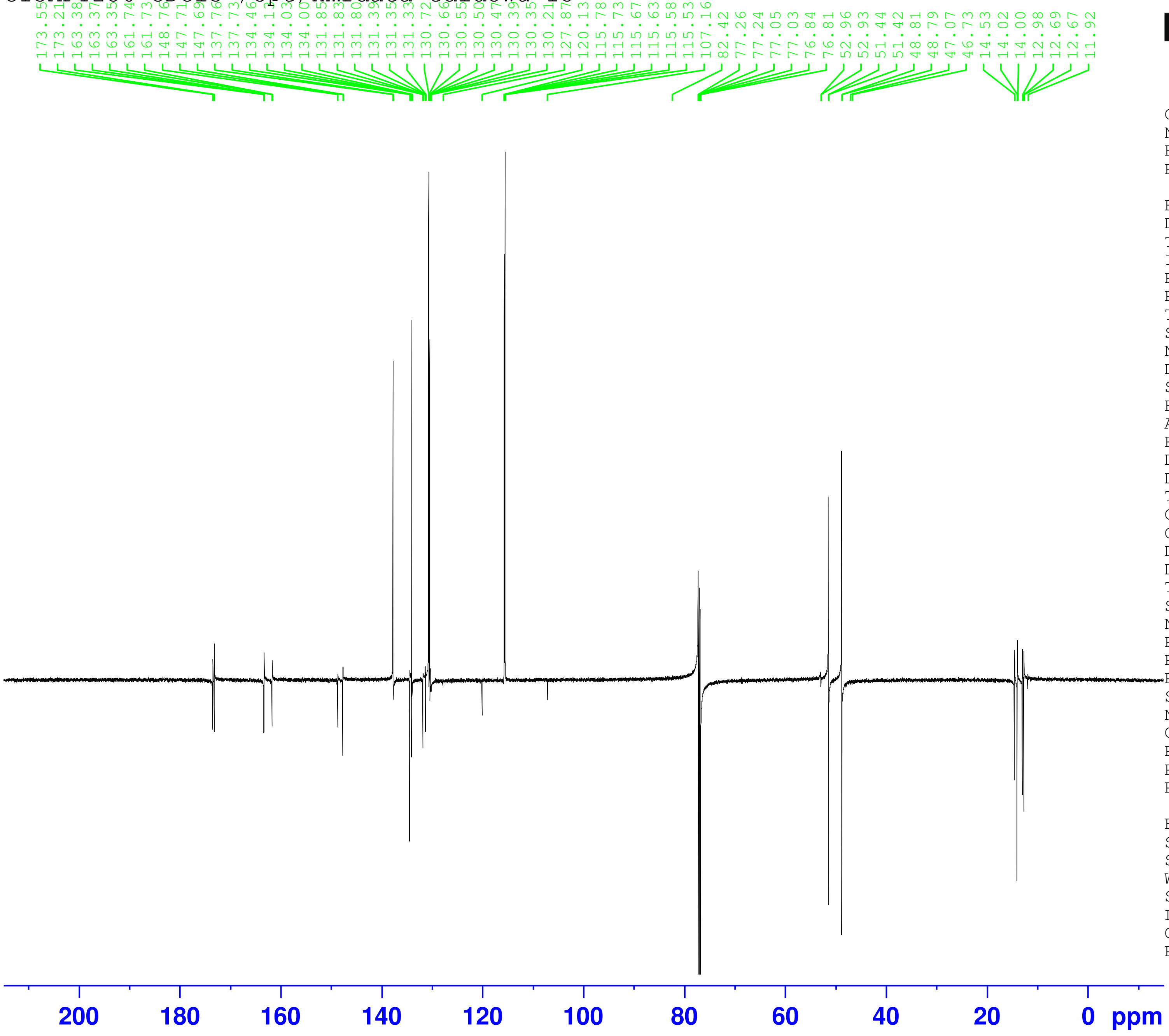

BRUKER

```
Current Data Parameters
NAME        TDI01074-clean
EXPNO                    9
PROCNO                   1

F2 - Acquisition Parameters
Date_             20240208
Time                 15.53 h
INSTRUM              spect
PROBHD      Z168773_0053 (
PULPROG         jmod.acqt0
TD                   65536
SOLVENT              CDCl3
NS                     256
DS                       4
SWH              34722.223 Hz
FIDRES            1.059638 Hz
AQ               0.9437184 sec
RG                    1030
DW                  14.400 usec
DE                   18.88 usec
TE                   298.1 K
CNST2          145.0000000
CNST11           1.0000000
D1              5.00000000 sec
D20             0.00689655 sec
TD0                      1
SFO1           150.9656791 MHz
NUC1                   13C
P1                   10.00 usec
P2                   20.00 usec
PLW1           88.38300323 W
SFO2           600.3224013 MHz
NUC2                    1H
CPDPRG[2           waltz16
PCPD2                80.00 usec
PLW2           21.85499954 W
PLW12           0.48474309 W

F2 - Processing parameters
SI                   32768
SF             150.9505840 MHz
WDW                     EM
SSB      0
LB                    1.00 Hz
GB       0
PC                    1.40
```
